# Bardet-Biedl and Alström syndromes in Spain

**DOI:** 10.1186/2046-2530-1-S1-P107

**Published:** 2012-11-16

**Authors:** D Valverde, T Piñeiro-Gallego, I Pereiro

**Affiliations:** 1 University of Vigo, Spain

## 

Bardet-Biedl (BBS, OMIM 209900) and Alström (AS, OMIM #203800) syndromes belong to a group of heterogenic disorders called ciliopathies where alterations on the cilia and the ciliary mechanisms are implicated. The genetically heterogeneous nature of BBS, with fifteen genes identified (BBS1-BBS15) to date, is also shown in the considerable inter- and intra-familial variation in the phenotype. Mutation screening of the involved genes has resulted in the identification of approximately 70% of the causative mutations, indicating that additional BBS genes have to be identified. *ALMS1* is the only gene associated with the development of AS. *ALMS1* encodes a novel protein, widely expressed and with unknown molecular function. We recruited 81 BBS families with 105 affected patients (44 females/ 61 males) and 5 AS families with 5 patients (4 females/ 1 male). Molecular analysis was performed using three strategies: homozygosity mapping when the family was consanguineous, BBS genotyping chip (Asper Ohthalmics), and direct sequencing. We were able to detected at least one mutation in 47% of the BBS patients. The allelic implication for the major genes in our patients was as follows: *BBS1* (56%), *BBS10* (20%), *BBS12* (11%), *BBS6* (7%), *BBS3* (4%), *BBS2* (1%) and *BBS5* (1%). In AS patients we could detect the two responsible mutations in two families, and only one mutation in heterozygous state in a third family. The molecular study of these syndromes can be very helpful in providing a diagnosis in this patients, hence appropriate genetic counselling for families and adequate medical follow-up for affected children.

